# Increase in Nontuberculous Mycobacteria Isolated in Shanghai, China: Results from a Population-Based Study

**DOI:** 10.1371/journal.pone.0109736

**Published:** 2014-10-16

**Authors:** Jie Wu, Yangyi Zhang, Jing Li, Senlin Lin, Lili Wang, Yuan Jiang, Qichao Pan, Xin Shen

**Affiliations:** Department of TB Control, Shanghai Municipal Center for Disease Control and Prevention, Shanghai, China; Fundació Institut d’Investigació en Ciències de la Salut Germans Trias i Pujol. Universitat Autònoma de Barcelona. CIBERES, Spain

## Abstract

**Background:**

In China, the prevalence of nontuberculous mycobacteria (NTM) in isolates from mycobacterial culture-positive patients with pulmonary tuberculosis (TB) is largely unknown.

**Methods:**

We used conventional biochemical and 16S rRNA gene sequencing to identify species of mycobacteria in specimens from patients suspected of having TB. Drug-susceptibility testing was performed on NTM isolates using the proportion method. We also determined the independent risk factors associated with infection with NTM compared with infection with *Mycobacterium tuberculosis*.

**Results:**

The overall rate of NTM isolated from mycobacterial culture-positive patients was 5.9% in this population, with a significantly increasing trend from 3.0% in 2008 to 8.5% in 2012 (*P* for trend <0.001). The organism most frequently identified was *M. kansasii* (45.0%), followed by *M. intracellulare* (20.8%) and *M. chelonae/abscessus* (14.9%). The overall proportion of isolates resistant to the four first-line anti-TB agents were 64.6% for isoniazid, 77.6% for streptomycin, 63.3% for rifampicin and 75.1% for ethambutol. The risk factors most often associated with NTM infection were older age (*P* for trend <0.001), being a resident of Shanghai (adjusted odds ratio [aOR], 1.48; 95% CI, 1.10–2.00), having been treated for tuberculosis (aOR, 1.64; 95% CI, 1.18–2.29), having a cavity on chest X-ray (aOR, 1.51; 95% CI, 1.16–1.96), and being sputum smear–negative (aOR, 1.59; 95% CI, 1.16–2.18).

**Conclusions:**

The prevalence of NTM isolated in Shanghai increased between 2008 and 2012, thus clinicians should consider NTM as a possible cause of TB-like disease. Accurate species identification is imperative so that proper treatment can be administered for diseases caused by the diversity of NTM species.

## Introduction

Mycobacteria other than *Mycobacterium tuberculosis* complex and *M. leprae* are known as nontuberculous mycobacteria (NTM) [1]–[3]. NTM show great diversity and are ubiquitous in the environment. These organisms are important opportunistic pathogens that have received much attention owing to an increase in the isolation of NTM from infections worldwide [4]–[9], especially in Asia [10]–[13]. NTM infections can cause clinical symptoms similar to tuberculosis (TB), which is caused by *M. tuberculosis*; however, the majority of NTM species are resistant to the most frequently used anti-TB agents. Therefore, it is possible that more patients with samples testing positive for acid-fast bacilli (AFB) have received unnecessary empirical anti-TB treatment.

A number of studies of the prevalence of pulmonary NTM infections in North America, Europe and Asia have been published recently [12]. It has been reported that the distribution of the species of NTM isolated from clinical specimens differs by continent and by country within continents [14], [15]. In the United States and Japan, *M. avium* complex and *M. kansasii* are the most common species [15], [16], while in England the most common is *M. kansasii*, and in Wales it is *M. malmoense* [17].

China has a high burden of TB, and although the prevalence of NTM associated disease is reported to be on the rise in Asia, few studies have reported the isolation rate of NTM, and related infectious diseases in China. A sentinel surveillance study showed that the prevalence of NTM among patients with suspected pulmonary TB in rural eastern China was low (1.6%) and stable [18]. However, mycobacterial culture and identification of NTM are not routinely performed in most areas of China. The epidemiology of NTM infections in China remains unclear.

If the factors associated with NTM infection could be identified, this could be used to quickly distinguish NTM from TB clinically, and help public health officials make decisions regarding contact investigations. A limited number of hospital-based studies have investigated the characteristics of NTM pulmonary diseases [19], [20]. However, few population-based studies have compared the differences in characteristics of patients with pulmonary TB and those infected with NTM.

In this study, we report the trends and diversity of NTM species in isolates from pulmonary samples from patients suspected of having TB. We also identify the characteristics associated with NTM infections in Shanghai, China. To our knowledge, this is the first population-based study to report on the epidemiology of NTM infection in China.

## Methods

### Study population

In Shanghai, patients suspected of having TB (e.g., cough lasting ≥2 weeks, or abnormalities identified by X-ray, or both) are referred to designated TB hospitals for confirmation and diagnosis. Three sputum samples, taken at different times, are collected from each individual referred to a TB hospital and tested by microscopy to detect AFB and by culture for *Mycobacterium* on Löwenstein-Jensen medium. For this study, isolates of *Mycobacterium* were sent to the Shanghai Municipal Center for Disease Control and Prevention for species identification and drug-susceptibility testing.

### Species identification

Conventional biochemical and 16S rRNA gene sequencing were used for species identification of mycobacteria. First, testing was performed with the growth inhibiting para-nitrobenzoic acid (PNB) to differentiate *M. tuberculosis* complex from NTM. Growth of *M. tuberculosis* complex is inhibited by PNB, whereas NTM are resistant. Second, NTM species were further identified by sequencing 16S rRNA with primers as described elsewhere [21]. Chromosomal DNA used as templates for amplification was extracted using the boiling lysis method. The 16S rRNA sequences were analyzed using Blast and the Ribosomal Differentiation of Medical Microorganisms (RIDOM) database (http://www.ridom-rdna.de/).

### Drug susceptibility testing

All mycobacterial isolates were tested for drug susceptibility against rifampicin (RFP), isoniazid (INH), streptomycin (SM), and ethambutol (EMB) using the proportion method on Löwenstein-Jensen medium at the following concentrations: 40 µg/mL, 0.2 µg/mL, 4 µg/mL, and 2 µg/mL, respectively.

### Statistical analysis

We used data from the Shanghai Municipal Centers for Disease Control and Prevention’s TB Information System to determine the risk factors associated with isolation of NTM; randomly selected TB patients from the same system were used as a reference group. The analysis included demographic information (age, sex, and area of residence), clinical information (TB history and cavitation shown on X-ray), and bacterial characteristics (results of sputum smears and drug-susceptibility testing).

Statistical analyses were performed using Stata (version 10.1/SE, StataCorp., College Station, Texas, USA). An initial analysis compared the possible risk factors associated with infection of NTM with data from TB patients using the χ^2^ test of proportions or Fisher’s exact test, as appropriate. To identify factors independently associated with the outcome variable, we performed a multiple logistic regression analysis. We also used a χ^2^ test to identify the trend in the proportion of NTM isolated from cultures during the study period. A *P* value of <0.05 was considered statistically significant.

### Ethics Statement

The study was approved by the Ethical Review Committee at the Shanghai Municipal Centers for Disease Control and Prevention. Since this was a retrospective study and all patients’ information used in this study had been routinely collected through the mandatory notification system, the requirement for informed consent was waived by the ethics committee.

## Results

### The increasing trend in NTM infection

**Figure 1:**
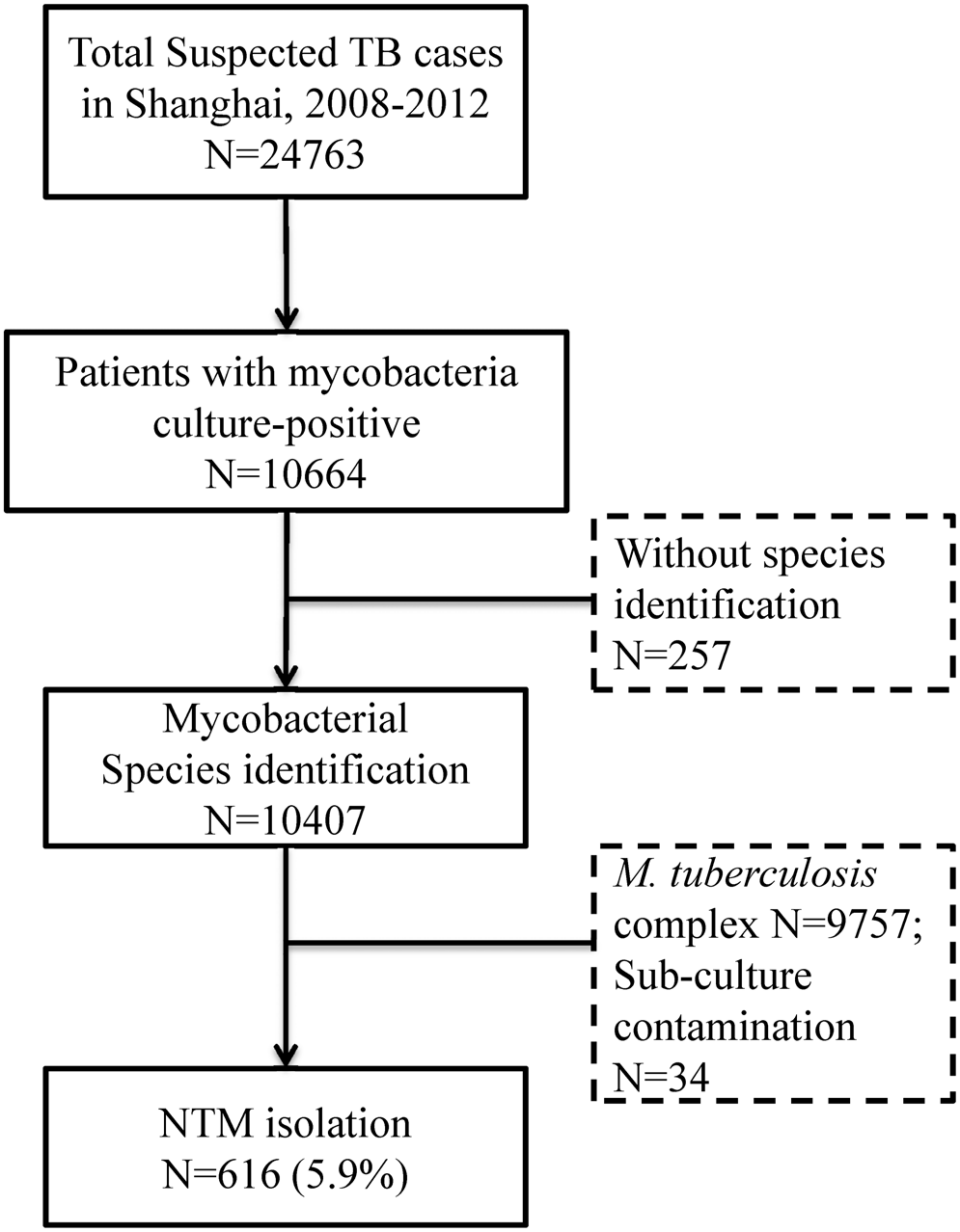
Isolation of nontuberculous mycobacteria from mycobacterial culture-positive patients in Shanghai, 2008–2012. MTB, *M. tuberculosis*; NTM, nontuberculous mycobacteria.

From January 2008 to December 2012, 24,763 suspected cases of TB were reported in Shanghai. Of these, 10,664 (43.1%) were culture-positive, including 10,407 from which the species of mycobacteria could be identified. A total of 650 isolates were identified as NTM using the biochemical method. Of these, 34 were contaminated and the species could not be identified; the remaining 616 (5.9%) samples were successfully subcultured and submitted for confirmation of NTM and detailed species identification (Figure 1). From 2008 to 2012, the isolation rate for NTM increased significantly, from 3.0% of cultures (95% confidence interval [CI], 2.3–3.9%) in 2008 to 8.5% (95% CI, 7.4–9.7%) in 2012 (*P* for the trend <0.001, Table 1).

**Table 1:** Prevalence of nontuberculous mycobacteria in samples from patients in Shanghai by year, 2008–2012.

No. of patients with	2008		2009		2010		2011		2012		Total	
Suspected TB cases	5737		5130		4774		4652		4470		24763	
Culture positive	2005		1910		2262		2137		2350		10664	
Species identification	1937		1875		2234		2118		2243		10407	
NTM isolates (%)	58	(3.0)	108	(5.8)	143	(6.4)	117	(5.5)	190	(8.5)	616	(5.9)
RGM	8	(13.7)	10	(9.3)	24	(16.8)	17	(14.5)	61	(32.1)	120	(19.5)
SGM	48	(82.7)	85	(78.7)	108	(81.2)	88	(75.2)	108	(56.8)	437	(70.9)
Others	2	(3.4)	13	(12.0)	11	(8.3)	12	(10.3)	21	(11.1)	59	(9.6)

Abbreviations: TB, tuberculosis; NTM, nontuberculous mycobacteria; RGM, rapidly growing mycobacteria; SGM, slowly growing mycobacteria.

### Species identification of NTM isolates

Table 2 shows detailed information about the species identified in the 616 NTM isolates. In all, 25 NTM species were identified. The most frequent organism identified was *M. kansasii* (45.0%), followed by *M. intracellulare* (20.8%), *M. chelonae*/*abscessus* (14.9%), *M. fortuitum* (4.5%), and *M. avium* (3.6%).

**Table 2:** Distribution of nontuberculous mycobacteria species by year, Shanghai, 2008–2012.

Year	Total	RGM				SGM						Others	
		*M. chelonae/abscessus*		*M. fortuitum*		*M. kansasii*		*M. intracellulare*		*M. avium*			
2008	58	4	(6.9)	4	(6.9)	32	(55.2)	15	(25.9)	1	(1.7)	2	(3.4)
2009	108	6	(5.6)	4	(3.7)	37	(34.3)	34	(31.5)	4	(3.7)	23	(12.0)
2010	143	15	(10.5)	9	(6.3)	78	(54.5)	26	(18.2)	4	(2.8)	11	(7.7)
2011	117	12	(10.3)	5	(4.3)	63	(53.8)	23	(19.6)	2	(1.7)	12	(10.3)
2012	190	55	(28.9)	6	(3.2)	67	(35.3)	30	(15.8)	11	(5.8)	21	(11.1)
Total	616	92	(14.9)	28	(4.5)	277	(45.0)	128	(20.8)	22	(3.6)	59	(9.6)

Abbreviations: NTM, nontuberculous mycobacteria; RGM, rapidly growing mycobacteria; SGM, slowly growing mycobacteria.

### Drug resistance profiles of NTM isolates

Of the 616 NTM isolates with results from drug-susceptibility testing, the overall proportion of isolates resistant to the four first-line anti-TB agents were 64.6% for INH, 77.6% for SM, 63.3% for RFP and 75.1% for EMB. Resistance to first-line anti-TB agents exceeded 85% in patients with *M. chelonae*/*abscessus* or *M. fortuitum* (Table 3).

**Table 3:** Results of in vitro testing for drug resistance for the five most prevalent species of nontuberculous mycobacteria, Shanghai, 2008–2012.

NTM species	No. (%) of isolates with resistant to*							
	INH		SM		RFP		EMB	
*M. kansasii* (n = 277)	151	(54.5)	185	(66.8)	150	(54.2)	198	(71.5)
*M. intracellulare* (n = 128)	86	(67.2)	113	(88.3)	72	(56.3)	106	(82.8)
*M. chelonae/abscessus* (n = 92)	83	(90.2)	87	(94.6)	80	(86.9)	82	(89.1)
*M. avium* (n = 22)	13	(59.1)	19	(86.4)	19	(86.4)	20	(90.9)
*M. fortuitum* (n = 28)	26	(92.9)	27	(96.4)	20	(71.4)	23	(82.1)
Total (n = 547)	359	(65.6)	431	(78.8)	341	(62.3)	429	(78.4)

*INH, isoniazid; SM, streptomycin; RFP, rifampicin; EMB, ethambutol.

### Risk factors for NTM infection

Based on information routinely collected by the TB registration system for the 616 patients with NTM isolated from their specimens, 465 (75.5%) were male, with a mean age of 54 years (range, 6–96 years); 474 (77.1%) were newly diagnosed cases; and 141 (22.9%) had a history of being treated for TB. A total of 427 (69.4%) were sputum smear-positive, and 261 (40.8%) had a cavity on chest X-ray. Table 4 shows the risk factors associated with NTM infection. Compared with the group of randomly selected TB patients, those who were infected with NTM were more likely to be older (*P* for trend <0.001), be local residents in Shanghai (adjusted odds ratio [aOR], 1.48; 95% CI, 1.10–2.00), have been treated for TB (aOR, 1.64; 95% CI, 1.18–2.29), have a cavity on chest X-ray (aOR, 1.51; 95% CI, 1.16–1.96), and be sputum smear-negative (aOR, 1.59; 95% CI, 1.16–2.18).

**Table 4:** Comparison of patients with isolates of *Mycobacterium tuberculosis* and those with nontuberculous mycobacteria, Shanghai, 2008–2012.

Variables	TB		NTM		Crude OR(95% CI)	p value	Adjusted OR(95% CI)†	p value
	No.	(%)	No.	(%)				
Sex								
Female	158	(25.2)	151	(24.5)	1.00			
Male	458	(74.8)	465	(75.5)	1.06 (0.81–1.39)	0.64		
Age, years						<0.01*		<0.01*
<30	242	(39.3)	85	(13.8)	1.00		1.00	
30–44	139	(22.6)	102	(16.6)	2.09 (1.46–2.98)	<0.01	2.08 (1.44–3.01)	<0.01
45–59	130	(21.1)	183	(29.7)	4.00 (2.87–5.60)	<0.01	3.31 (2.26–4.86)	<0.01
60–74	62	(10.0)	126	(20.5)	5.78 (3.91–8.56)	<0.01	4.90 (3.16–7.60)	<0.01
≥75	43	(7.0)	120	(19.5)	7.94 (5.18–12.2)	<0.01	6.73 (4.15–10.93)	<0.01
Resident registration								
Migrants	292	(47.4)	146	(23.7)	1.00		1.00	
Residents	314	(50.1)	458	(74.5)	2.92 (2.26–3.76)	<0.01	1.48 (1.10–2.00)	0.01
TB history								
New case	540	(87.6)	474	(77.1)	1.00		1.00	
Treated case	76	(12.3)	141	(22.9)	2.11 (1.56–2.87)	<0.01	1.64 (1.18–2.29)	<0.01
Positive sputum smear								
Yes	451	(73.2)	427	(69.4)	1.00		1.00	
No	121	(19.6)	126	(20.5)	1.10 (0.83–1.46)	0.50	1.59 (1.16–2.18)	<0.01
Unknown	44	(7.1)	62	(10.1)	1.48 (0.99–2.24)	0.05	2.59 (1.55–4.30)	<0.01
Cavitary chest radiograph								
No	320	(52.0)	280	(48.7)	1.00		1.00	
Yes	241	(39.1)	261	(40.8)	1.23 (0.97–1.57)	0.07	1.51 (1.16–1.96)	<0.01
Unknown	55	(8.9)	129	(10.5)	1.53 (1.04–2.26)	0.02		

Abbreviations: TB, tuberculosis; NTM, nontuberculous mycobacteria; OR, odds ratio; CI, confidence interval.

*P value for trend.

† Adjusted variables are shown in the table.

## Discussion

To our knowledge, this is the first large-scale study in Shanghai to estimate the prevalence of NTM using comprehensive laboratory analysis. The overall prevalence of NTM in Shanghai is 5.9%, with a significant increasing trend. Genetic sequencing showed that the most prevalent NTM species in Shanghai was *M. kansasii*, which accounted for nearly half of all NTM species isolated. The independent risk factors associated with NTM infection were older age, being a local resident, having a history of TB, being sputum smear-negative and having a cavity on chest X-ray.

The increase in NTM identified during this five-year period may be the result of an increase in the prevalence of infections caused by NTM. It could also have resulted from an increased awareness of these bacteria as human pathogens and improved methods for identifying NTM. During the past decades, the worldwide increase in the proportion of NTM isolated from cultures has been associated with the HIV epidemic and immunodeficiency in the host. Although we did not know the HIV infection rate in the study population, the prevalence of HIV infection in Shanghai is relatively low [28]. Additionally, other risk factors, including the number of elderly people, may have contributed to the increase in NTM isolated from samples in Shanghai.

The prevalence of mycobacteria species responsible for different diseases varies markedly by geographic region. Despite reports of a rise in the isolation of NTM in Asia, the most prevalent NTM species were *M. avium* complex. In a study from Taiwan, *M. avium* complex accounted for most NTM disease, followed by *M. chelonae*/*abscessus* and *M. fortuitum*. A meta-analysis also found that *M. avium* complex is the most prevalent cause of NTM disease in East Asia [14]. In China, until recently there have been few reports of NTM disease or isolates: one study in Shandong reported that 80% of clinical NTM isolates were *M. intracellulare* [18]; in Guangzhou, during 1994–2003 a study of 136 NTM isolates found that the majority of species identified were *M. avium* complex and *M. chelonae/abscessus* [22]. In Shanghai, one hospital-based study that included 248 NTM isolates from Shanghai Pulmonary Hospital found that between 2005 and 2008 the most prevalent species were *M. chelonae*/*abscessus* (26.7%), *M. fortuitum* (15.4%), *M. kansasii* (14.2%) and *M. intracellulare* (13.1%) [20]. The result from the hospital-based study in Shanghai was different from what we observed in the current study. The differences can be attributed to differences in sampling, with our results representing the population-based prevalence of NTM isolated in Shanghai city.

It is important to identify NTM isolates to the species level because each NTM species has its own clinical significance and antimicrobial susceptibility. Most NTM species were resistant to the first-line anti-TB agents used for treatment of TB; therefore, misdiagnosis of NTM infection could lead to inappropriate anti-TB treatment. Although in Shanghai we cultured all specimens from patients suspected of having TB and performed PNB testing, a period of 1–2 months was required to confirm NTM. Generally, patients with sputum smear-positive specimens are presumed to be infected with *M. tuberculosis* and are treated with anti-TB agents based on the clinician’s experience. Currently, there are no guidelines for performing in vitro drug-susceptibility testing for different NTM species. Thus, the appropriate treatment of NTM disease can be recommended based only on the results of local surveillance for NTM and the resistance profiles of the species.

In this study, the most prevalent NTM species in Shanghai was *M. kansasii*, which had been previously identified in water systems and can persist for up to 12 months; however, this species is difficult to isolate from soil and water [23]. It has been reported that a high prevalence of *M. kansasii* is always associated with water pollution [24]–[26]. In the current study, *M. kansasii* accounted for nearly half of the NTM species isolated; further investigation is needed to determine whether this observation is associated with contamination of water systems [27].

One limitation of our study was the lack of identification of true disease caused by NTM infection. In clinical practice, it is difficult to determine whether the isolation of NTM from sputum specimens means that the patient has NTM pulmonary disease [15]. Two or more positive sputum cultures with the same NTM strains are highly predictive of NTM pulmonary disease [8], [9]. However, in most TB laboratories in Shanghai, it is hard to obtain a second specimen from patients after the long wait for confirmation using the culture method.

## Conclusion

Because the prevalence of NTM isolates has increased in Shanghai, clinicians there should consider NTM as a possible cause of TB-like symptoms. Accurate species identification is imperative so that proper treatment can be determined for diseases caused by the diversity of NTM species. Further study is needed to investigate the possible epidemiological links between specific NTM species (e.g., *M. kansasii*) isolated from environmental sources with those isolated from patients’ specimens.
